# Editorial: Functional assessments of the ocular circulation

**DOI:** 10.3389/fmed.2023.1222022

**Published:** 2023-06-08

**Authors:** Rebekka Heitmar, Dietmar Link, Konstantin Kotliar, Doreen Schmidl, Sascha Klee

**Affiliations:** ^1^Centre for Vision Across the Lifespan, School of Applied Sciences, University of Huddersfield, Huddersfield, United Kingdom; ^2^Division Optoelectrophysiological Engineering, Department of Computer Science and Automation, Institute of Biomedical Engineering and Informatics, Technische Universität Ilmenau, Ilmenau, Germany; ^3^Medical Engineering and Technomathematics, Aachen University of Applied Sciences, Aachen, Germany; ^4^Department of Clinical Pharmacology, Medical University of Vienna, Vienna, Austria; ^5^Division Biostatistics and Data Science, Department General Health Studies, Karl Landsteiner University of Health Sciences, Krems an der Donau, Austria

**Keywords:** retinal vessel, imaging, vasculature, ocular, systemic, methodology, technology

In recent years, ocular vascular functional imaging has gained importance due to the increasing availability of new methods such as optical coherence tomography angiography (OCTA) or Doppler OCT (DOCT). In contrast to static (morphological) imaging, functional imaging has the advantage that it provides insight into physiological and pathophysiological processes, which may greatly benefit the diagnosis, monitoring, and treatment of ocular diseases associated with vascular dysfunction. In addition, functional parameters are usually quantitative, which also makes them attractive as potential endpoints in clinical studies (1).

Changes in ocular vascular parameters have also been shown to be associated with changes in the cerebral vasculature and therefore might have a great potential to become biomarkers for cerebrovascular diseases in the future (2, 3). Studies also found that retinal vascular parameters can assist in cardiovascular risk assessment (4).

The technologies and methodologies for functional assessment of the ocular vasculature (i.e., vessel diameter, blood flow, branching angles, vessel density/tortuosity; as well as their dynamic changes) allow for direct observation of the retinal and intra-retinal vascular beds at rest but also when challenged. Flicker light provocation, cold exposure, inhalation of gas mixtures, exercise, and others are only few interventions that have been employed to study the functional aspects of the retinal circulation. All of those have advanced our understanding of physiological processes, but also of alterations due to systemic and local disease processes and aging.

This issue entitled “*Functional assessments of the ocular circulation*” will provide an insight into methodological, technical, analytical, and clinical aspects of ocular imaging techniques and their potential in assessing steady-state and functional aspects of ocular and systemic disease. While the analyses of “big data” and the utilization of “artificial intelligence” has already seen a sharp increase in the implementation of retinal vascular parameters in such analyses (5, 6), it is still important to keep an eye on technical and methodological factors which can impact retinal vascular parameterisation prior to their use in further processing such as realized by artificial intelligence.

Böhm et al. provide a well-structured review of the available methods to assess blood flow and vascular reactivity in the retina. The authors offer an overview of clinical studies in humans and in experimental animals. They start with a brief essay on ocular diseases with impaired blood flow involvement following with a detailed description of the measuring methods. Summarizing classical techniques, such as laser Doppler velocimetry and flowmetry (7), as well as retinal oximetry (8), described in detailed reviews elsewhere, the authors provide an in-depth digest on modern high-potential retinal vascular imaging methods that got widespread applications in the past decade, e.g., OCTA, DOCT, laser speckle flowmetry, *in-vivo* red blood cell labeling and *ex-vivo* transmitted light microscopy.

The present review has a stronger focus on techniques directly measuring or related to ocular blood flow but less so on static and dynamic retinal vascular analysis methodologies which one can find elsewhere (9–11). Other promising methods, such as OCT-based velocimetry (12), adaptive optics with rtx1 (13), laser interferometry, confocal scanning laser Doppler flowmetry (7) are not described in detail here and may be of interest for those seeking further suitable methods to assess the retinal vasculature.

Schanner et al. analyzed fundus images from a long-term diabetic retinopathy follow-up study to identify the limits and opportunities of quantitative comparability of geometric retinal vessel properties, such as the vessel diameter and other derived parameters. Their work focused on the fact that these images come from changing imaging modalities, such as different generations of equipment, or imaging settings (e.g., image centering, resolution, viewing angle, illumination wavelength). Analog images were included by scanning the original. They then investigated the relationship of image conversion factor and image centration on retinal vessel geometric characteristics. In summary, Schanner et al. emphasize the high clinical relevance by showing that there are possibilities to use image data obtained in clinical routine of the same eye from different modalities and settings, e.g., from different devices, device generations, and with different image properties for a long-term analysis. On the way to standardization in the calculation and determination of quantitative retinal vascular parameters, this work can provide an important step in data preparation and suitability testing (14, 15).

Dong et al. investigated the role of astrocytes in ocular blood flow regulation during hyperoxia. For this purpose, the authors conducted an *in-vitro* and an *in-vivo* study to assess the expression of vasoactive substances within rat lamina cribrosa tissue and optic nerve head astrocytes. In their experiments they confirmed that nitric oxide synthase (NOS), endothelin-1 (ET-1) and components of the renin-angiotensin system (RAS) are expressed by ONH astrocytes. These findings point toward an important role of astrocytes in ocular blood flow regulation, since several previous studies in humans have confirmed that these substances are involved in ocular blood flow control (16, 17). These observations could lay ground for future studies in eyes with diseases which are known to impair blood flow regulation as described by Böhm et al. in this issue. In addition, recent evidence has accumulated that astrocytes appear to play a crucial role in the pathophysiology of glaucoma and diabetic retinopathy (18–21). The findings of Dong et al. could therefore provide a possible link between disturbed blood flow regulation and retinal astrocytes in ocular diseases.

Wang and Xia explored the role of macular vascular parameters as measured by OCTA in young myopic individuals and their relation to visual function. Structural differences of the retinal superficial vasculature and layer thickness parameters between myopic and non-myopic individuals have been previously reported (22, 23) and highlighted regarding their potential implication in the development of future pathology. However, some of the reasons for differences in vessel density and dimensional measures such as the foveal avascular zone (FAZ) are likely due to magnification effects (24). Nonetheless, visual function changes in young subjects with myopia may be linked to structural alterations of the retinal layers including vascular parameters. Structural vascular changes may potentially precede functional loss and are important considering the increased risk of myopic patients developing glaucoma (25). In fact, a recent study by Hong et al. showed that ocular hemodynamic parameters were linked with central visual function (26) in patients with glaucoma and myopia. While there is limited data on long-term progression and interaction, it shows the importance of considering ocular vascular parameters when assessing myopic individuals.

Fan et al. conducted a systematic review and meta-analysis to provide an overview on Behcet's disease without ocular manifestations. Behcet's disease was first described in 1937 by Huluci Behçet and is classified as a vasculitis (27). This systematic review identifies a significant enlargement of the FAZ and decrease of the parafoveal vascular density as measured by OCTA prior to other ocular manifestations. While their study included high quality trials therefore having a low risk of bias, there is still potential latent bias due to differences in OCT imaging devices (RTVue XR Avanti or Spectralis OCT), macular scan sizes (3^*^3 mm or 6^*^6 mm) and hence slightly distinct calculations of the same parameters in different studies. Their results further identified the need for more long-term observations to corroborate their findings of the potential use of OCTA vascular parameter as clinical markers in Behcet's disease. Besides following up on structural vascular parameters, such as vessel density, it would be useful to assess retinal vascular dynamics including neurovascular coupling response in Behcet's disease to identify if such pathological mechanisms are compromised already at a subclinical level (28). In addition, the analyses of the retinal arteriolar and venous microcirculation could potentially be applicable for differential diagnostic purposes as in Behcet's disease the systemic involvement appears to be predominantly of venous origin (29). As it is currently not possible to separate arteriolar and venular vessels using conventional OCTA, this could be an avenue for future studies exploring retinal vascular involvement in Behcet's disease in detail.

Tornow et al. developed a multi-color video ophthalmoscope and investigated its technical properties for measuring the absorption of blood. The new device is based on a monochromatic video-ophthalmoscope also developed by the authors. This uses a highly sensitive complementary metal-oxide-semiconductor (CMOS) sensor and an organic light-emitting diode (OLED) display in the aerial image as a fixation target to reduce the subject's eye movements during video recording. Tornow et al. replaced the illumination LED by a 45-degree mirror at the position of an image of the pupil plane. A white LED placed outside the optical axis of the video-ophthalmoscope is imaged on to the 45-degree mirror. By inserting narrow bandpass filters between the white LED and the mirror, different wavelengths can be selected for illumination. Manipulating the spectral and spatio-temporal properties of the illumination path of a fundus camera to obtain additional functional properties of the visual system is also known from other works (30, 31). In this technical setting, however, the authors were able to show that the wavelength dependency of the pulsatile absorption amplitude follows the spectral distribution of the light absorption of blood. Based on the authors conclusions this suggests that the pulsatile light intensity changes are likely to be caused by light absorption of changing blood volume and are not due to mechanical changes of the eyeball caused by the pulsating intraocular pressure.

In summary, there are several different methods, technologies and analytical approaches on a wide variety of structural and functional vascular parameters derived from retinal imaging. Collectively, each of the methodologies enable a wide range of mechanistic and clinical examinations. However, the lack of standardized protocols is one of the main stumbling blocks limiting the conclusions that can be drawn for clinical purposes. Employing standardized imaging protocols will be the first step which will subsequently enable the curation of larger datasets across sites (and research groups) to facilitate identification of the most potent biomarkers derived from the retinal vasculature.

## Author contributions

All authors listed have made a substantial, direct, and intellectual contribution to the work and approved it for publication.
